# Integrating multi-omics and experimental techniques to decode ubiquitinated protein modifications in hepatocellular carcinoma

**DOI:** 10.3389/fphar.2025.1545472

**Published:** 2025-04-11

**Authors:** Haikun Yang, Yuan Chen, Zheng Zhou, Yanjing Wang, Peng Li, Yang Li

**Affiliations:** ^1^ Department of Gastroenterology, Shanxi Provincial People’s Hospital, Taiyuan, China; ^2^ Department of Geriatric Medicine, Shanxi Bethune Hospital, Shanxi Academy of Medical Sciences, Third Hospital of Shanxi Medical University, Tongji Shanxi Hospital, Taiyuan, China; ^3^ Department of Geriatrics, Tongji Hospital, Tongji Medical College, Huazhong University of Science and Technology, Wuhan, China; ^4^ The Hepatobiliary and Pancreatic Disease Area Department of Shanxi Provincial People Hospital, Taiyuan, China

**Keywords:** UBE2C, ubiquitination, tumor cells, immune regulation, prognosis, tumor microenvironment

## Abstract

**Background:**

Ubiquitination, a critical post-translational modification, plays a pivotal role in regulating protein stability and activity, influencing various aspects of cancer development, including metabolic reprogramming, immune evasion, and tumor progression. However, the specific role of ubiquitination in hepatocellular carcinoma (HCC), particularly in relation to the tumor microenvironment (TME), remains poorly understood. This study aims to systematically explore the role of ubiquitination in shaping the TME of HCC, with a focus on its impact on cancer progression and immune modulation.

**Methods:**

We performed bioinformatics analysis by integrating multiple publicly available HCC datasets to assess the ubiquitination status across various cell types in the TME, including plasma cells, fibroblasts, endothelial cells, and epithelial-mesenchymal transition (EMT) cells. Ubiquitination scores were calculated to categorize these cell types, and survival data, along with spatial transcriptomics, were employed to evaluate how different levels of ubiquitination influence HCC progression. *In vitro* experiments, such as transwell, CCK8, and wound healing assays, were used to further investigate the role of the key ubiquitination gene UBE2C in HCC phenotypes.

**Results:**

Our study revealed that ubiquitination-related genes are significantly upregulated in HCC tissues, with high expression levels correlating with poor prognosis in patients. Pathway analysis showed that these genes are enriched in key processes such as cell cycle regulation, DNA repair, metabolic reprogramming, and p53 signaling. These pathways contribute to the TME by promoting tumor cell proliferation, facilitating matrix remodeling, and enhancing angiogenesis. Notably, UBE2C, a critical ubiquitination enzyme, appears to play a key role in immune evasion, potentially by inhibiting anti-tumor immune responses and reducing the immune system’s ability to recognize and eliminate tumor cells. Furthermore, experimental data confirmed that UBE2C overexpression promotes HCC cell proliferation, invasion, and metastasis, further supporting its role in tumor progression and TME remodeling.

**Conclusion:**

This study reveals the multifaceted regulatory roles of ubiquitination in HCC. Ubiquitination not only supports proliferation and anti-apoptotic functions within tumor cells but also promotes tumor progression by modulating the activity of immune and stromal cells. Among all ubiquitination-related genes, UBE2C emerges as a potential prognostic biomarker and therapeutic target in HCC, offering new directions for precision treatment of HCC in the future.

## 1 Introduction

Hepatocellular carcinoma (HCC) is one of the most prevalent malignant tumors worldwide, characterized by a high incidence and mortality rate (Yang et al., 2014; Xiong et al., 2024; Lu et al., 2023). According to the World Health Organization (WHO), HCC ranks as the fourth leading cause of cancer-related deaths globally, accounting for over 800,000 deaths annually, with particularly high incidence rates in Asia and Africa (Bray et al., 2018). The primary risk factors for HCC include chronic infections with hepatitis B virus (HBV) and hepatitis C virus (HCV), long-term alcohol abuse, and fatty liver disease (El-Serag, 2012; Friedman et al., 2018). Due to the frequent late-stage diagnosis of HCC, treatment outcomes are often poor, with high recurrence rates and a persistently low 5-year survival rate.Consequently, research into early diagnostic methods and novel therapeutic strategies for HCC is of critical importance (Comprehensive and Integrative Genomic Characterization of Hepatocellular CarcinomaCancer Genome Atlas Research Network, 2017; Fan T. et al., 2024).

Ubiquitination is a prevalent post-translational modification in which ubiquitin molecules are covalently attached to target proteins, thereby regulating their stability, activity, and cellular localization (Sun et al., 2020; Sun and Zhang, 2022). This process typically involves a cascade of reactions orchestrated by E1 ubiquitin-activating enzymes, E2 ubiquitin-conjugating enzymes, and E3 ubiquitin ligases (Zhang and Jiang, 2021). Ubiquitination plays key roles in various biological processes, including cell cycle regulation, DNA repair, and signal transduction. Meanwhile, deubiquitinating enzymes (DUBs) can reverse this process by removing ubiquitin, maintaining protein homeostasis. An imbalance in ubiquitination and deubiquitination can lead to the development of various diseases, including cancer (Zhang et al., 2023; Chen Y. et al., 2024).

In recent years, growing evidence has highlighted the critical role of ubiquitination in the initiation and progression of hepatocellular carcinoma (HCC) (Gong et al., 2024; Liu Z. et al., 2024; Zhao et al., 2024). For instance, MDM2, a key E3 ligase, regulates the degradation of p53 via ubiquitination, thereby affecting HCC cell proliferation and apoptosis (Shi and Gu, 2012). Furthermore, certain deubiquitinating enzymes (DUBs), such as USP7 and USP10, influence HCC cell growth by modulating cell cycle and apoptosis-related proteins (Li et al., 2023; Li and Liu, 2020; Henningsen et al., 2021). Dysregulated ubiquitination in HCC is not only closely associated with the malignant biological behaviors of tumors but also contributes to resistance against anticancer drugs, further complicating treatment.Given its pivotal role in HCC, ubiquitination is increasingly regarded as a potential therapeutic target (Chang and Ding, 2018). However, its impact on the tumor microenvironment (TME) of HCC remains poorly understood. Therefore, elucidating the intrinsic link between ubiquitination and alterations in the HCC tumor microenvironment is essential for advancing precision treatments for HCC (Lv et al., 2020).

## 2 Materials and methods

### 2.1 Cell culture

Human hepatocellular carcinoma (HCC) cell lines, including Huh7 and Hep3B, were cultured in Dulbecco’s modified Eagle’s medium (DMEM; HyClone) supplemented with 10% fetal bovine serum (FBS; Hyclone), 100 U/L penicillin, and 100 mg/L streptomycin (Thermo Fisher), at 37°C in a 5% CO_2_ environment. Lipofectamine 3000 (Invitrogen, Carlsbad, CA, United States) was employed for transfection of Negative Control (NC) and DKC1 siRNA (RiboBio, Guangzhou, China) into the HCC cells, following the manufacturer’s instructions.

### 2.2 shRNA knockdown

Plasmids expressing shRNA, specifically designed to target UBE2C, were carefully constructed with the assistance of GenePharma. During cultivation, the cells were treated with viral supernatants and polybrene (Sigma Aldrich) in the culture medium. After 24 h of incubation, the cells were transferred to fresh medium containing 2.0 μg/mL of puromycin. The efficiency of UBE2C knockdown was confirmed 2 days later using qRT-PCR analysis.

### 2.3 qPCR assay

Total RNA extraction was carried out utilizing the RNA Eazy Fast Tissue/Cell Kit (TIANGEN Biotech) in accordance with the manufacturer’s guidelines. Subsequently, cDNA synthesis was performed using the FastKing RT Kit (TIANGEN Biotech), adhering to the provided protocol. Real-time PCR analysis was conducted with the application of the SuperReal PreMix Plus (TIANGEN Biotech) reagent, implemented on the StepOnePlus Real-Time PCR System. The PCR reaction encompassed an initial pre-denaturation phase at 95°C for 15 min, followed by 40 amplification cycles, comprising denaturation at 95°C for 10 s, annealing at 72°C for 20 s, and extension at 60°C for 20 s. Primer sequences utilized were procured from Sangon Biotech. (Species of Human Origin) UBE2C Forward Primer: 5′-GAC​CTC​TCC​TTG​TTG​CTG​CC-3′, reverse primer 5′-GTC​CAG​GTC​ATT​GGG​CTG​AC-3'; PCR signals 2-44^−ΔΔCT^ was used to calculate the expression of genes mRNA levels. The following sequences were used: 5′-CCT​CTC​CTT​GTT​GCT​GCC​G-3′ for human UBE2C shRNA.

### 2.4 Transwell assay

Cell migration and invasion of HCC cells were evaluated using the Transwell assay. Briefly, 5 × 10^4 cells were seeded into Transwell chambers coated with Matrigel (BD Biosciences, San Jose, CA) for invasion or uncoated for migration. The upper chamber was filled with serum-free medium, while the lower chamber contained complete DMEM medium. After 24 h of culture, the cells that had migrated or invaded through the membrane were fixed with 4% paraformaldehyde and stained with 0.1% crystal violet. Cell numbers were subsequently quantified using a light microscope (Thermo Fisher, Waltham, MA, United States).

### 2.5 CCK-8 assay

Cell viability was assessed using the Cell Counting Kit-8 (CCK8) assay. After 24 h of transfection, cells were seeded into 96-well plates at a density of 2500 cells per well in 100 µL of complete medium and incubated at 37°C. Following each experiment, 10 µL of CCK8 reagent (Beyotime, Shanghai, China) was added to each well, and the cells were further incubated for 4 h at 37°C. The optical density value (OD450) was then measured using a microplate reader.

### 2.6 Wound healing assay

The migratory behavior of Huh7 and Hep3B cells was analyzed using a wound healing assay, which offered detailed observations of their movement patterns. Cells, post-transfection, were grown in a six-well plate and maintained at 37°C until they reached about 80% confluence. A sterile 200 μL pipette tip was then utilized to make a precise linear scrape through the layers of cells to establish a uniform wound. Subsequent to this, the wells were washed twice with phosphate-buffered saline (PBS) to remove any detached cells, and the medium was replaced with serum-free medium. The closure of the wound was observed and documented at 0 h and 24 h using an inverted microscope (Olympus, Japan), allowing for measurement of the migration distance covered by the cells across the wound area.

### 2.7 Clonogenic formation

600 cells were seeded in 6-well plates. These plates were then placed in the incubator for 14 days until clones formed, each consisting of at least 50 cells. Subsequently, the colonies were stained using a 0.1% crystal violet solution.

### 2.8 Protein expression and immunohistochemistry

We used the CTPAC database to validate the difference in the expression of UBE2C protein in hepatocellular carcinoma tissues and normal liver tissues. The expression levels of UBE2C in hepatocellular carcinoma tissues and normal tissues were verified by immunohistochemical sections from the HPA database.

### 2.9 Data sources

This study utilized single-cell sequencing data from the GEO database (https://www.ncbi.nlm.nih.gov/geo/), specifically the dataset GSE149614, which contains sequencing data from 10 hepatocellular carcinoma (HCC) patients. We selected non-tumor and primary tumor samples for analysis. Spatial transcriptomics data were obtained from the primary HCC tissue section GSM6177612. Additionally, RNA-seq data for pancreatic cancer, comprising 424 samples and associated survival data, were acquired from the TCGA cohort via the UCSC Xena platform (https://xena.ucsc.edu/) for survival analysis. We retrieved a set of 78 ubiquitination-related genes from the GO database (https://geneontology.org/).

### 2.10 Quality control, dimensionality reduction, clustering, and cell type identification

After importing the raw single-cell sequencing data, we performed initial processing using the Seurat package (version 4.3.0), including quality control, dimensionality reduction, and visualization (Hao et al., 2021). To ensure data reliability, we applied stringent quality control criteria, selecting cells with gene expression levels between 500 and 6000 and mitochondrial gene expression below 15%. The data were normalized and standardized using the NormalizeData and ScaleData functions, followed by principal component analysis (PCA) with the RunPCA function for dimensionality reduction. To integrate data from different sources, we used the Harmony package (version 1.2.0) for batch effect correction. The top 20 principal components were then selected for clustering at a resolution of 0.3, resulting in 17 cell clusters. Based on liver tissue marker genes from the Cellmarker website and differential expression analysis for each cell cluster using the FindAllMarkers function, we categorized cells into three main types: hepatocytes, stromal cells, and immune cells. Subsets were extracted and re-processed with similar steps to refine subcluster identification, followed by a second round of cell annotation to achieve a final classification (Gu et al., 2024).

### 2.11 Ubiquitination scoring

Using the 78 ubiquitination-related genes retrieved from the GO database, we applied five algorithms—AUCell, UCell, singscore, ssGSEA, and AddModuleScore—to score ubiquitination levels in the single-cell dataset. Scores were standardized using the scale function and normalized using the normalize function to ensure comparability across methods, yielding a comprehensive ubiquitination score for each cell. Based on median ubiquitination scores, cells were divided into high and low ubiquitination groups, focusing on hepatocytes, plasma cells, fibroblasts, endothelial cells, and effector memory T cells (Hao et al., 2021; Yu et al., 2012).

### 2.12 Cell-cell communication analysis and copy number variation (CNV) analysis

To explore cell interactions within the HCC tumor microenvironment, we conducted cell-cell communication analysis using the CellChat package, involving ligand-receptor matching, network construction, signaling pathway analysis, hierarchical and centrality analysis (Jin et al., 2021). This revealed interaction mechanisms and differences between high and low ubiquitination cells and other cell types. We performed CNV analysis using the copykat function from the CopyKAT package to predict cellular malignancy (Chi et al., 2022).

### 2.13 Differential expression analysis

We used the FindMarkers function to analyze gene expression differences between tumor and normal tissues, retaining only upregulated genes. By intersecting differentially expressed genes with the ubiquitination gene set, we identified ubiquitination-related differentially expressed genes. To investigate differences between high and low ubiquitination cells, we visualized differential gene expression between these groups (Chi et al., 2023).

### 2.14 Enrichment analysis

To investigate functional differences between cells with varying ubiquitination levels, we conducted GO and KEGG enrichment analyses. Genes upregulated in high-ubiquitination cells, identified using the FindMarkers function, were used for enrichment analysis. The clusterProfiler package facilitated retrieval of gene sets from GO, KEGG, and GSEA databases and visualized the results. Additionally, the GSVA package, combined with the HALLMARK gene set, was used to identify tumor-related biological processes. For functional enrichment, we used the bitr function from clusterProfiler to convert gene symbols to ENTREZ IDs, then applied the compareClusterfunction to perform KEGG enrichment, revealing functional impacts of ubiquitination on hepatocytes within the tumor microenvironment (Yu et al., 2012).

### 2.15 Metabolic analysis

Metabolic states were analyzed using the scMetabolism package, generating a heatmap of metabolic scores. The AUCell method within the sc.metabolism.Seurat function assessed metabolic activity, with KEGG database-specified pathways for metabolic pathway enrichment. This analysis provided insights into the metabolic mechanisms and differences among cells, offering clues to their functional roles in the tumor microenvironment.

### 2.16 Evaluation of infiltration and prognostic analysis for cells with high and low ubiquitination levels

Using the FindMarkers function, we identified marker genes for cells with high and low ubiquitination levels and conducted ssGSEA scoring on TCGA data to classify patients into high and low infiltration groups. Survival analysis, using the survival and survminer packages, was performed to predict and evaluate prognostic differences based on infiltration levels. Survival curves were fitted using the survfit function, and Kaplan-Meier survival plots were generated with ggsurvplot to analyze the impact of infiltration levels on prognosis (Chen H. et al., 2024).

### 2.17 Deconvolution analysis of spatial transcriptomics data

Quality control was performed on spatial transcriptomics data using the Seurat package, excluding ribosomal and mitochondrial genes. After normalization with the SCTransform function and PCA-based dimensionality reduction, clustering with the top 20 principal components yielded 7 cell clusters (Luo et al., 2024). The scMetabolism package, in conjunction with the KEGG database, utilized the AUCell method to assess metabolic pathways of ubiquitination genes, exploring cellular functional characteristics. Spatial deconvolution was conducted with the SpaceXR package using annotated single-cell data to infer spatial distribution and analyze cell communication patterns, with spatial dependencies and cellular responses in pancreatic cancer tissues analyzed using the mistyR package (He et al., 2024).

### 2.18 Expression, prognostic, and clinical analysis of key ubiquitination gene UBE2C

After intersecting differential genes, we identified six key genes, with UBE2C emerging as the most significant through prognostic analysis. Differential expression analysis was performed on TCGA data, validated by three GEO datasets: GSE14520, GSE39791, and GSE54236. Based on UBE2C expression, patients were stratified into high and low expression groups for survival analysis, highlighting the prognostic impact of this pivotal ubiquitination gene in HCC. Kaplan-Meier survival curves were generated using data from multiple sources. Additionally, we conducted KM curve analysis to evaluate UBE2C expression in immune and stromal cells in relation to survival and clinical outcomes, complemented by KEGG enrichment analysis for UBE2C (Jiang et al., 2024).

### 2.19 Statistical analysis

All statistical analyses were conducted using R version 4.3.3 (64-bit) and associated packages. The Wilcoxon rank-sum test was used for differences between groups of continuous variables, and Spearman correlation analysis was used to assess correlations between variables. Statistical significance was set at P < 0.05.

## 3 Results

### 3.1 Data collection and quality control

**FIGURE 1:**
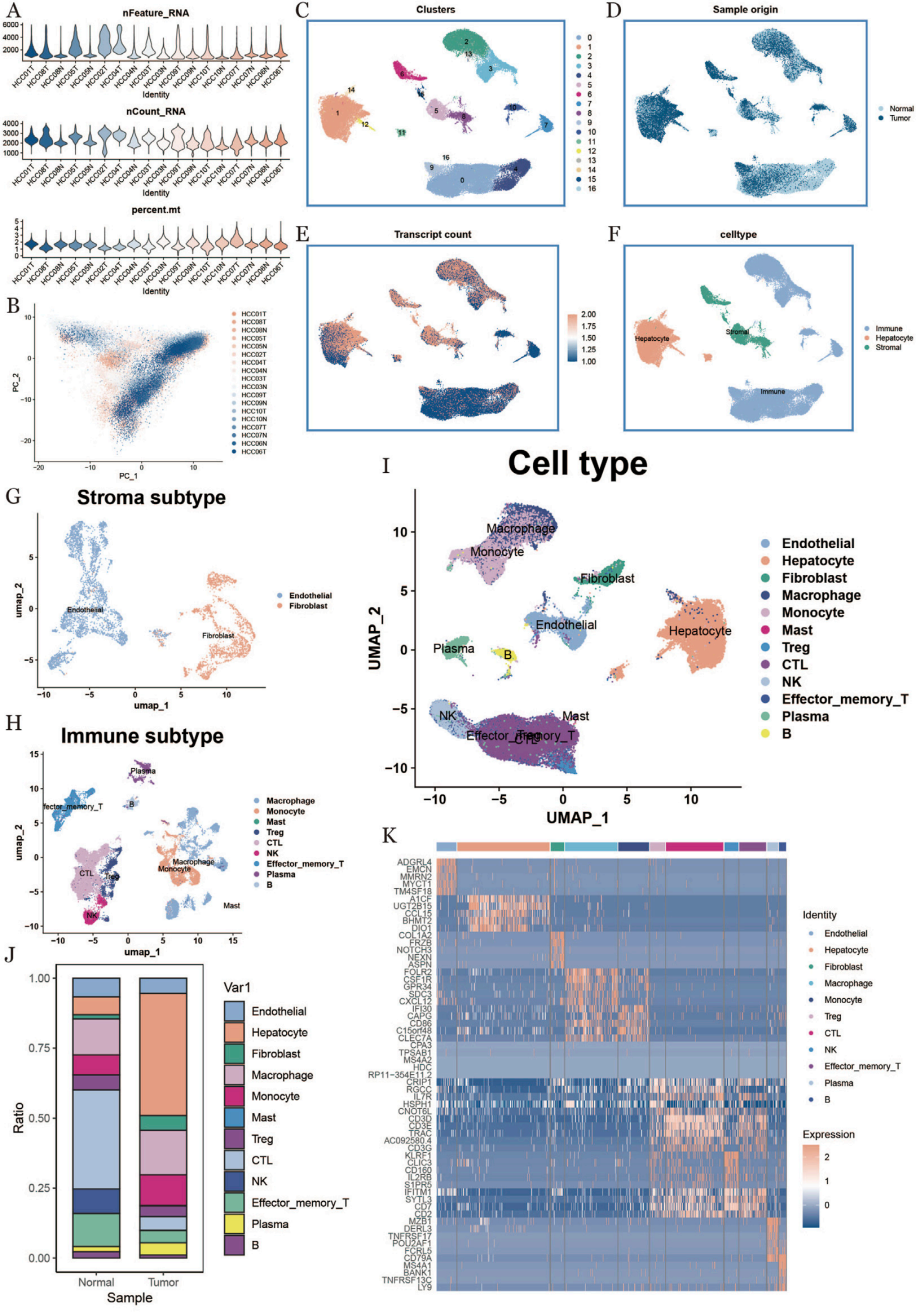
Single-cell data quality control, dimensionality reduction clustering, and cell type identification. **(A)**: Sample characteristic violin map after quality control, including gene number, count number, and mitochondrial proportion. **(B)**: Cell distribution map of different sample sources. **(C)**: Cell cluster distribution umap map. **(D)**: Cell distribution umap map of different tissue type sources. **(E)**: Characteristic expression of nCount_RNA umap plot. **(F)**: preliminary cell type identification result display plot, different colors represent different cell types. **(G)**: stromal cell subpopulation distribution umap plot. **(H)**: immune cell subpopulation distribution umap plot. **(I)**: final cell type identification result display plot. **(J)**: cell type percentage histogram. **(K)**: heatmap of genes with the top five expression by each cell type.

In this study, we analyzed single-cell transcriptomic data (ID: GSE149614) from the GEO database, comprising 18 tumor and corresponding normal liver tissue samples from 10 hepatocellular carcinoma (HCC) patients. To ensure high-quality single-cell data analysis, we implemented strict quality control measures across all samples. Various quality metrics, including UMI counts and the expression levels of mitochondrial and hemoglobin genes (Figure 1A), were assessed to exclude aging cells, erythrocytes, and mitochondrial signals. Additionally, the Harmony algorithm was applied to remove batch effects from sequencing, ensuring that the results reflected only biological differences between samples (Figure 1B).

Following dimensionality reduction and clustering, we applied UMAP to organize the 61,776 quality-controlled cells into 16 distinct clusters (Figure 1C). We also examined data distribution differences across samples (Figure 1D), between tumor and normal tissues (Figure 1E), and the density variation in mRNA expression (Figure 1F).Initial cell type identification was performed by detecting marker genes for specific cells using the “FindAllMarkers” function. Cells were classified based on marker gene expression patterns and the upregulation of genes within each cell cluster (Figure 1G). Figure 1H highlights the distribution differences of various cell types between tumor and normal tissues, while the marker genes for each cell cluster are visualized in a heatmap (Figure 1I).For abundant immune cell populations, including myeloid cells, B cells, and T/NK cells, we conducted sub-clustering analysis using a resolution of 0.1. This analysis identified subtypes such as plasma cells, CTLs, EMTs, Tregs, and macrophages (Figures 1J). Finally, we summarized the distribution differences of all identified cell types between tumor and normal tissues (Figure 1K).

### 3.2 Identification of high-ubiquitination cells

**FIGURE 2:**
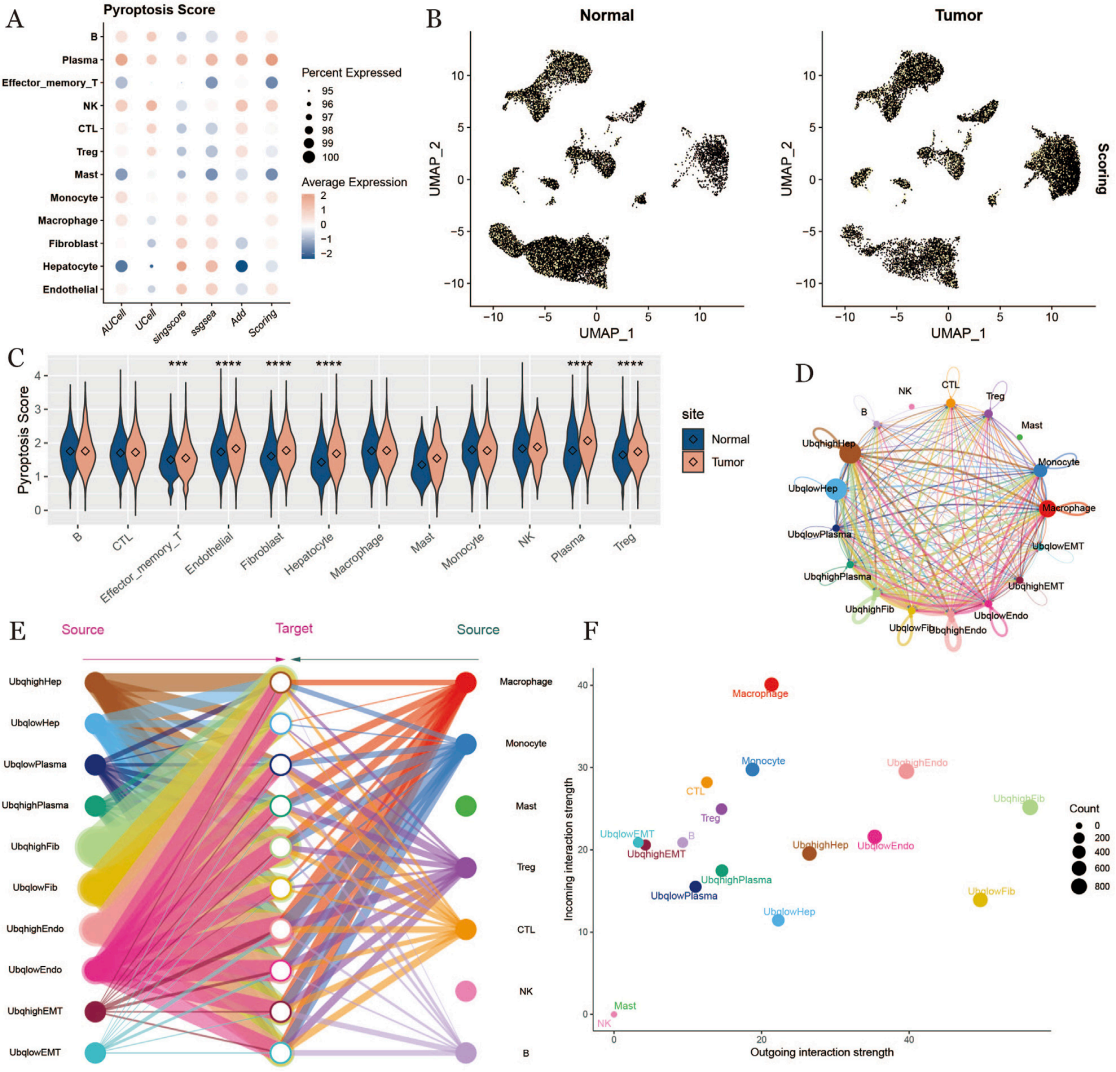
Ubiquitination scoring, cell communication. **(A)** ubiquitination scoring bubble plot, horizontal coordinate indicates scoring method, vertical coordinate indicates cell type, the lighter the color the higher the score. **(B)** umap plot of ubiquitination scoring results, displayed by tissue type. **(C)** violin plot of the difference between ubiquitination scores of the normal group and tumor group, where “****” indicates a p-value <0.001 and “****” indicates a p-value <0.0001, the more asterisks, the smaller the p-value, and the more significant the difference. **(D)**: Chordal plot of cellular communication based on ubiquitination level. **(E)**: Hierarchical plot of cellular communication. **(F)**: Scatter plot of intercellular signaling, with each point representing one kind of cell and the horizontal and vertical axes indicate the ability of that kind of cell to send and receive signals, respectively.

To identify cells with high expression of ubiquitination-related genes, we employed multiple scoring methods to evaluate ubiquitination levels across various cell types, including effector memory T cells, plasma cells, CTLs, Tregs, NK cells, macrophages, endothelial cells, hepatocytes, fibroblasts, and monocytes. The ubiquitination scores were visualized using UMAP plots for both normal and tumor samples (Figures 2A,B).Next, we performed a significance analysis of ubiquitination scores across different cell types under normal and tumor conditions. The results revealed that ubiquitination scores were significantly higher in tumor samples, suggesting a potential role for ubiquitination in tumor progression (Figure 2C).To further investigate ubiquitination characteristics within the HCC tumor microenvironment, we categorized cells into high- and low-ubiquitination groups based on their ubiquitination scores and conducted a cell communication analysis. High-ubiquitination hepatocytes (UbqhighHep) exhibited the strongest cellular communication signals (Figure 2D). Additionally, we visualized overall communication interactions between cells with differential ubiquitination, highlighting active interactions among endothelial cells and fibroblasts (Figure 2E). Finally, comparative analysis of communication signals across all cells revealed pronounced signaling between endothelial and fibroblast cells under high ubiquitination conditions (Figure 2F).

### 3.3 Ubiquitination in hepatocytes within the tumor microenvironment

**FIGURE 3:**
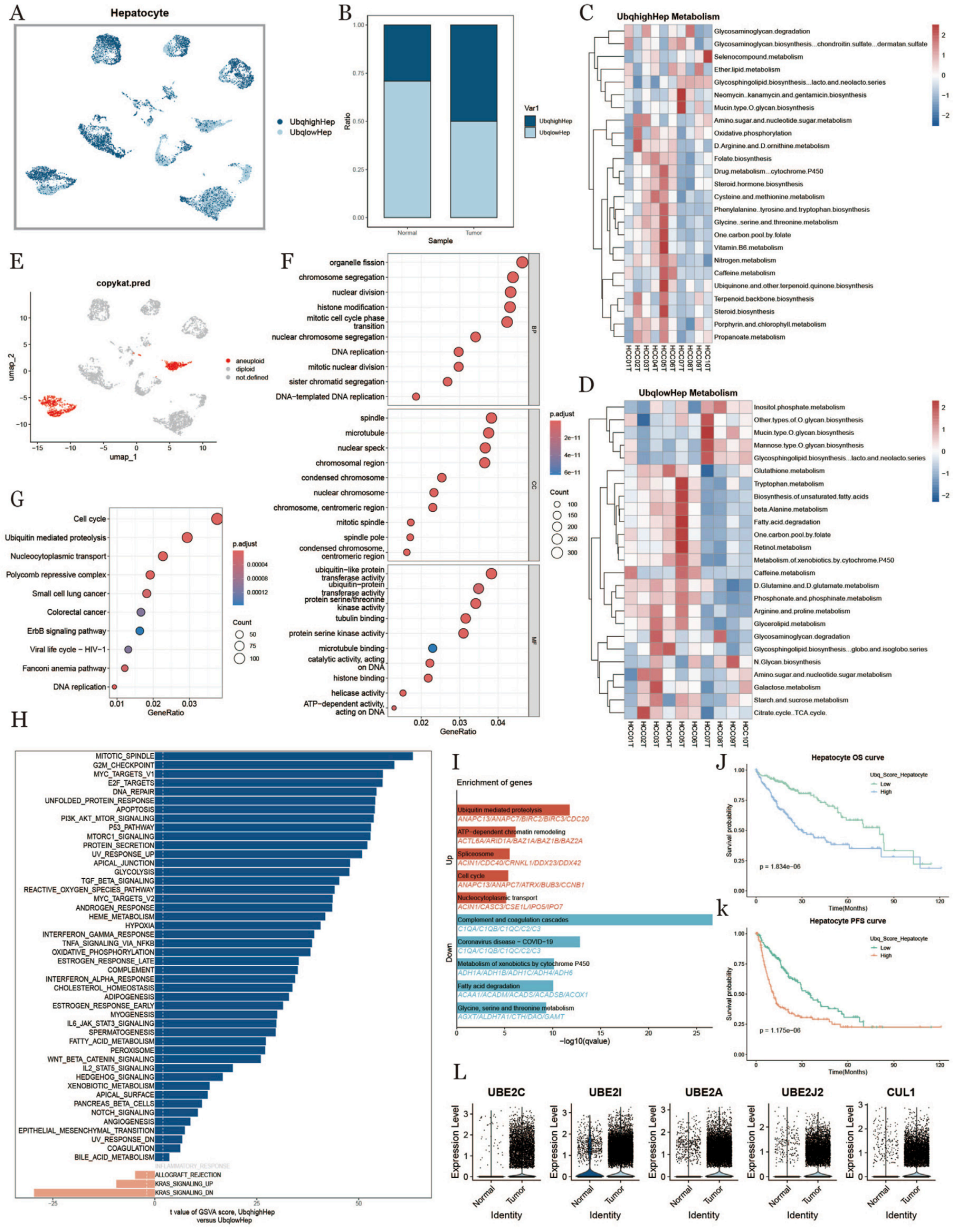
Hepatocyte characterization based on ubiquitination level Hepatocytes **(A)**: umap plot of the distribution of hepatocytes with high and low ubiquitination levels. **(B)**: Difference in the percentage of hepatocytes with high and low ubiquitination levels in normal and tumor tissues. **(C, D)**: Metabolic heatmap of hepatocytes with high and low ubiquitination levels. **(E)**: Hepatocyte copy number variability analysis results. **(F)**: GO enrichment analysis results plot. **(G)**: KEGG enrichment analysis result graph. **(H)**: Hepatocyte pathway comparison result between high ubiquitination level and low ubiquitination level in tumor tissues. **(I)**: Differential gene enrichment analysis of hepatocytes with high and low ubiquitination level. **(J, K)**: KM curves of overall survival and progression-free survival of hepatocytes under the difference of ubiquitination level. **(L)**: Differential genes related to ubiquitination of hepatocytes in normal tissues and tumor tissues.

To investigate ubiquitination characteristics in hepatocytes within the HCC tumor microenvironment, we divided hepatocytes into high- and low-ubiquitination groups based on ubiquitination scores (Figure 3A). We found that hepatocytes in tumor samples had higher ubiquitination levels compared to normal samples (Figures 3B,E). In Figures 3C,D, we compared metabolic differences between hepatocytes with different ubiquitination levels, discovering that metabolic pathways and cellular processes in hepatocytes were influenced by ubiquitination in liver cancer. Hepatocytes with high ubiquitination showed increased activity in metabolic pathways, such as amino acid metabolism and one-carbon metabolism. GO enrichment analysis indicated that hepatocytes with high ubiquitination scores were more active throughout the cell division process, with higher levels of proteolysis and translation (Figures 3F,I). Figure 3G presents the results of GSVA enrichment analysis, indicating that ubiquitination primarily affects the cell cycle. To explore heterogeneity between the two groups, we performed GSEA analysis, revealing functional differences in hepatocytes with high and low ubiquitination scores (Figure 3H). We observed that the high-ubiquitination group showed enhanced expression of cell cycle-related genes. These results suggest that ubiquitination likely influences proteins involved in proteolysis, affecting the normal cell cycle and leading to the transformation of normal cells into malignant ones in HCC development.To investigate the prognostic impact of ubiquitination in HCC, we further analyzed the expression levels of ubiquitination-related genes, finding that most genes were upregulated in HCC tissues (Figure 3L). Survival curves indicated that patients with high ubiquitination levels had shorter overall survival (OS) and progression-free survival (PFS) compared to those with low ubiquitination levels (Figures 3J,K).

### 3.4 Ubiquitination in plasma cells within the tumor microenvironment

**FIGURE 4:**
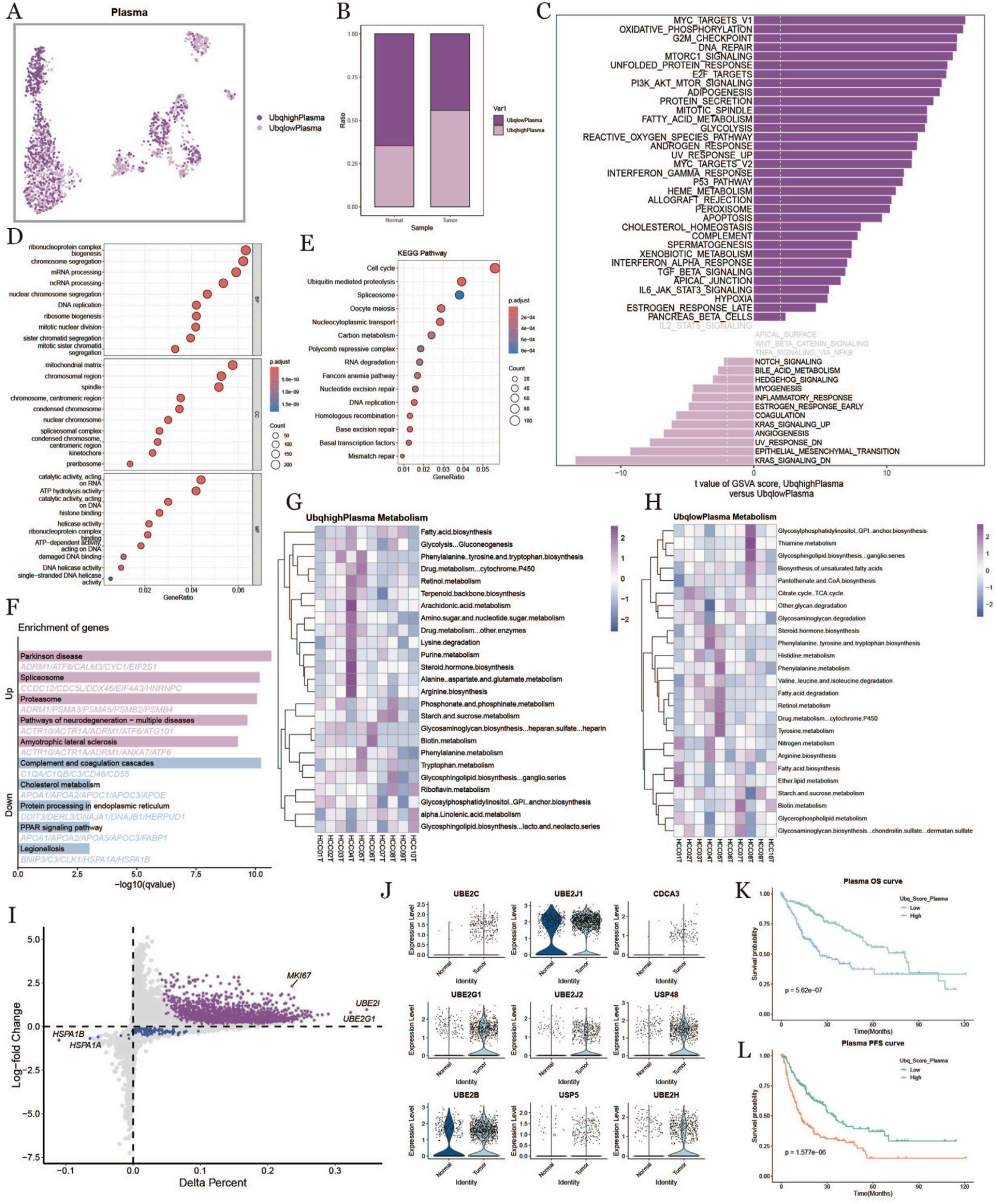
Plasma cell characterization based on ubiquitination level. **(A)**, umap plot of plasma cell distribution under the difference of ubiquitination level. **(B)**, plot of high and low ubiquitination scores into the percentage of fibroblasts in normal and tumor tissues. **(C)**, comparison of plasma cell pathways with high ubiquitination scores and low ubiquitination scores in tumor tissues. **(D)**, results of GO enrichment analysis. **(E)**, results of KEGG enrichment analysis. **(F)**, results of high and low ubiquitination score plasma cells differential gene enrichment analysis. **(G, H)**: metabolic heatmap of plasma cells with high and low ubiquitination levels. **(I)**: diagonal plot of differential genes of plasma cells with high *versus* low ubiquitination scores in tumor tissues. **(J)**: ubiquitination-associated differential genes of plasma cells in normal *versus* tumor tissues. **(K, L)**: km curves of overall and progression-free survival for plasma cells with high and low ubiquitination scores.

To study the ubiquitination characteristics of plasma cells in the HCC tumor microenvironment, we categorized plasma cells into high- and low-ubiquitination groups based on ubiquitination scores (Figure 4A). Plasma cells in the tumor group exhibited higher ubiquitination levels compared to normal samples (Figure 4B). Figure 4C shows the results of GSVA enrichment analysis, revealing that ubiquitination affected physiological processes related to metabolism, inflammation, and the cell cycle. GO enrichment and KEGG analysis showed that plasma cells with high ubiquitination scores were active in ribosome biogenesis, protein processing, and metabolic pathways (Figures 4D,F). Figure 4E presents the results of KEGG enrichment analysis, indicating that ubiquitination also significantly impacted the cell cycle. In Figures 4G,H, we compared metabolic differences in plasma cells under different ubiquitination states, finding that metabolic pathways and cellular processes in plasma cells were affected by ubiquitination in liver cancer. To examine the prognostic effect of ubiquitination on HCC, we further analyzed the expression levels of ubiquitination-related genes. Genes like MKI67, UBE2C, and UBE2I were upregulated in samples with higher ubiquitination levels, and ubiquitination-related genes were also widely upregulated in tumor samples (Figures 4I,J). Survival curves revealed that patients with high ubiquitination levels had shorter OS and PFS compared to those with low ubiquitination (Figure 4K, L).

### 3.5 Fibroblast ubiquitination in tumor microenvironment

**FIGURE 5:**
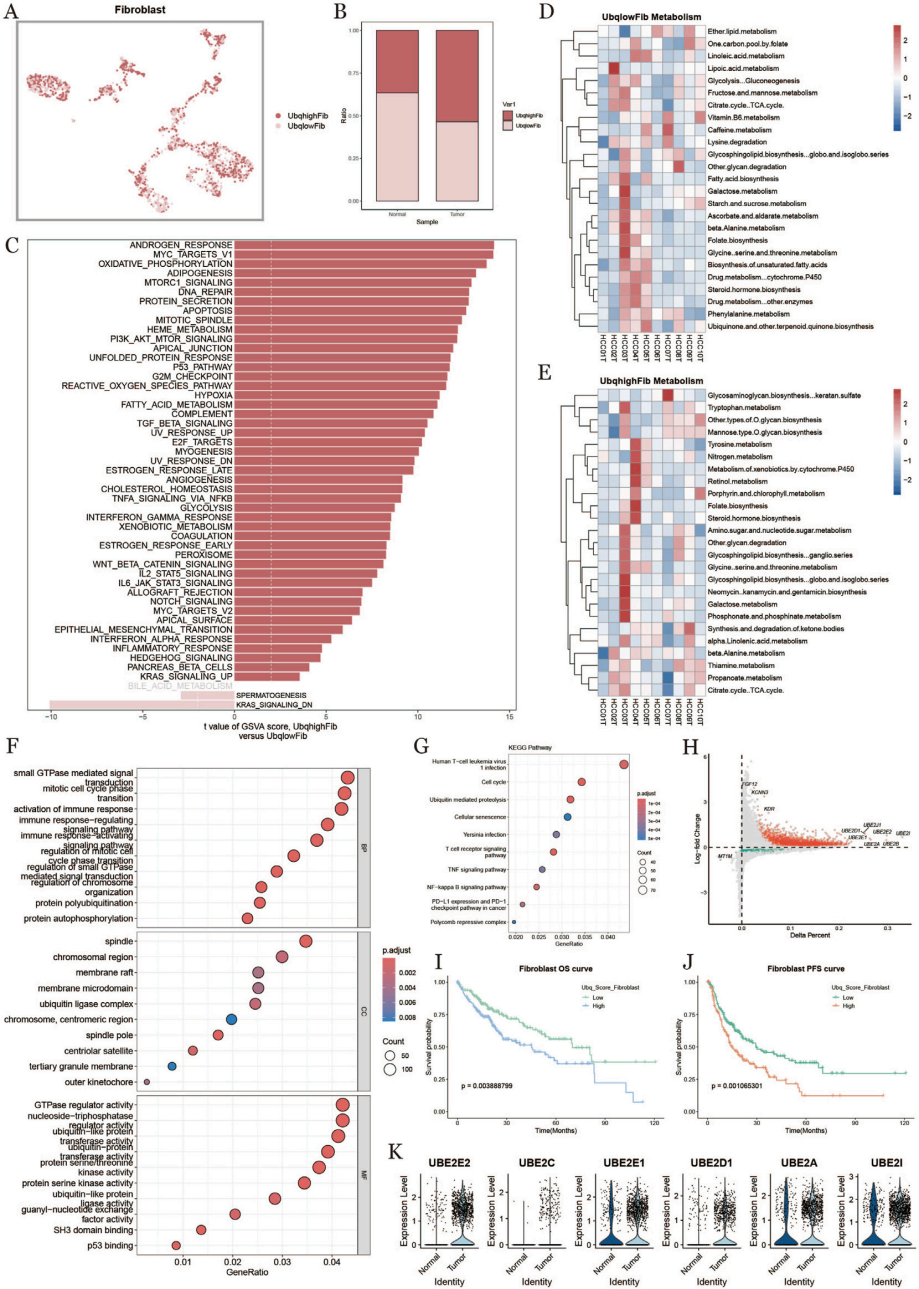
Fibroblast characterization based on ubiquitination level **(A)** umap plot of the distribution of fibroblasts with high and low ubiquitination levels. **(B)** plot of the percentage of fibroblasts with high and low ubiquitination levels in normal and tumor tissues. **(C)** comparison of fibroblast pathways in tumor tissues with high and low ubiquitination levels. **(D, E)** metabolic heatmap of fibroblasts with high and low ubiquitination levels. **(F)** GO functional enrichment analysis dot plot. **(G)** KEGG enrichment analysis dot plot. **(H)** Diagonal dot plot of fibroblast differential genes at high and low ubiquitination levels. **(I, J)** Survival KM curves of fibroblasts with overall survival and progression-free survival under ubiquitination differences. **(K)** Differential genes related to ubiquitination in fibroblasts in normal and tumor tissues.

To investigate the ubiquitination characteristics of fibroblasts in the liver cancer tumor microenvironment, we classified fibroblasts into high-expression (UbqhighFib) and low-expression (UbqlowFib) groups based on ubiquitination scores (Figure 5A). Fibroblasts in the tumor group exhibited significantly higher ubiquitination levels compared to the normal group (Figure 5B). Gene Set Variation Analysis (GSVA) enrichment analysis (Figure 5C) highlighted the role of ubiquitination in key biological processes, including oxidative phosphorylation and apoptosis. Metabolic analysis revealed that high-ubiquitination fibroblasts exhibited active metabolic pathways (Figures 5D,E). GO and KEGG pathway analyses showed that high-ubiquitination fibroblasts were involved in biological processes such as small GTPase signaling, immune response, protein autophagy, and chromatin regulation (Figure 5F). KEGG enrichment analysis (Figure 5G) identified significantly affected pathways in high-ubiquitination fibroblasts, including the cell cycle, ubiquitin-mediated protein degradation, and apoptosis.The volcano plot (Figure 5H) displayed the differential expression of ubiquitination-related genes between the high- and low-ubiquitination groups, with genes such as UBE2E2, UBE2C, and UBE2E1 showing significant upregulation in the high-ubiquitination group. Survival analysis revealed that patients with high-ubiquitination fibroblasts had shorter overall survival (OS) and progression-free survival (PFS), suggesting that high ubiquitination may be associated with poor prognosis in liver cancer patients (Figures 5I,J). Figure 5K shows that the expression levels of key ubiquitination-related genes (e.g., UBE2E2, UBE2C, UBE2E1, UBE2A) were significantly upregulated in the high-ubiquitination group.

### 3.6 Endothelial cell ubiquitination in tumor microenvironment

**FIGURE 6:**
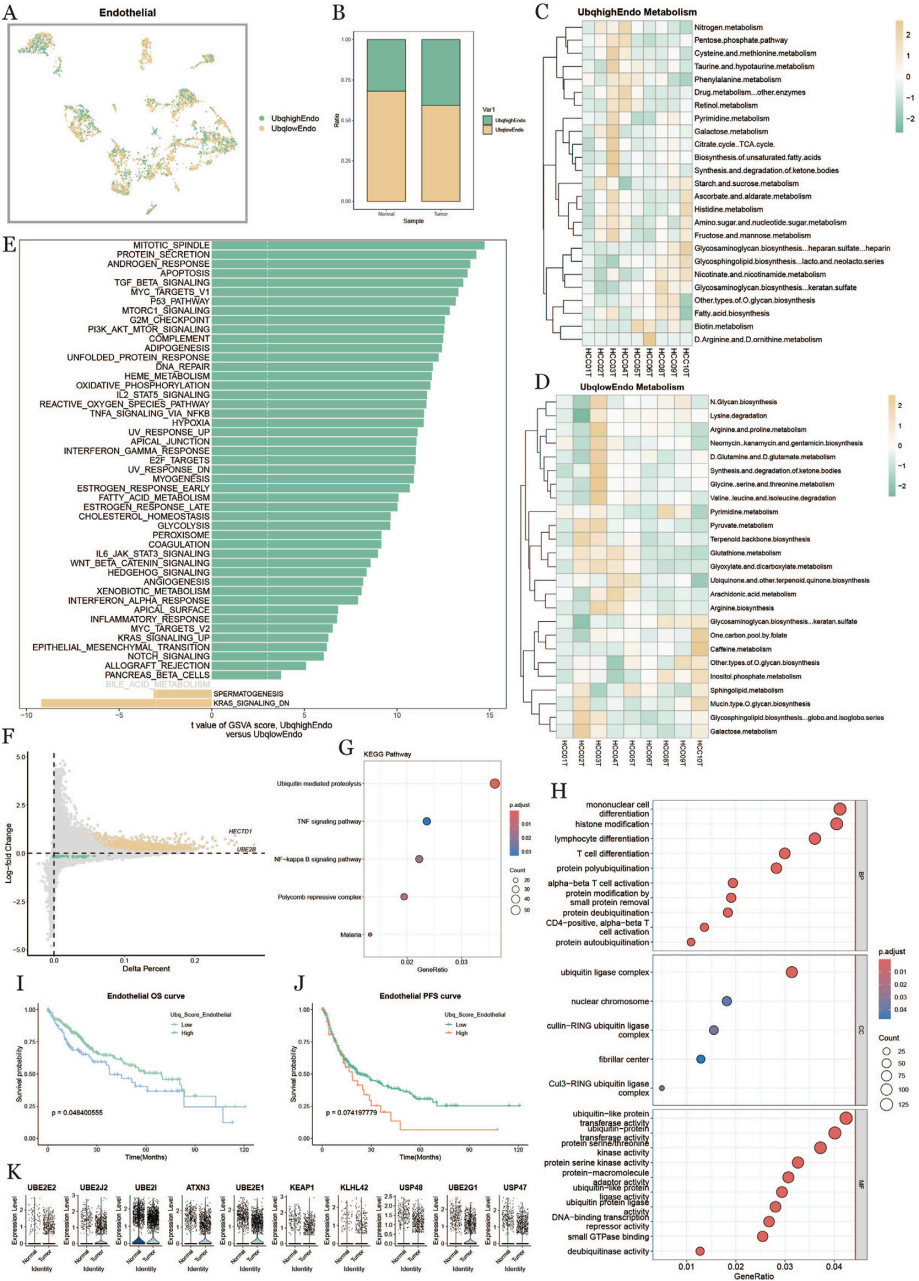
Endothelial cell characterization based on ubiquitination level. **(A)**: Distribution of endothelial cells based on differences in ubiquitination level. **(B)**: Percentage of endothelial cells in normal vs. tumor tissues under differences in ubiquitination level. **(C, D)**: Metabolic heatmap of endothelial cells with high and low ubiquitination scores. **(E)**: Comparison of endothelial cell pathways with high vs. low ubiquitination levels in tumor tissues. **(F)**: Comparison of endothelial cell pathways with high vs. low ubiquitination levels in tumor tissues. Level *versus* low ubiquitination level endothelial cell differential gene diagonal dot plot. **(G)**: KEGG enrichment analysis result plot. **(H)**: GO enrichment analysis result plot. **(I, J)**: Survival KM curves of endothelial cell overall survival and progression-free survival under the difference of ubiquitination level. **(K)**: Difference in expression of ubiquitinylation-related differential genes in endothelial cells in normal and tumor tissues plot.

To explore ubiquitination in endothelial cells within the HCC tumor microenvironment, we categorized endothelial cells into high-expression (UbqhighEndo) and low-expression (UbqlowEndo) groups based on ubiquitination scores (Figure 6A). Analysis showed that endothelial cells in tumors had higher ubiquitination levels compared to those in normal tissue (Figure 6B). Metabolic heatmaps (Figures 6C,D) displayed the enriched metabolic pathways in high and low ubiquitination endothelial cells, linking ubiquitination to metabolic activity in pathways like nitrogen metabolism, ketone synthesis, and amino acid metabolism. GSVA enrichment analysis (Figure 6E) demonstrated that ubiquitination influenced multiple biological processes and signaling pathways, including oxidative phosphorylation, cell cycle, inflammatory response, PI3K-Akt signaling, and TGF-beta signaling. The volcano plot (Figure 6F) indicated that genes like METTL7A were significantly upregulated in the high-ubiquitination group. KEGG pathway analysis (Figure 6G) showed significant enrichment in protein degradation, TNF signaling, and NF-kappa B signaling pathways. GO enrichment analysis (Figure 6H) further highlighted ubiquitination’s potential role in immune regulation and cell differentiation, affecting functions such as monocyte differentiation, histone modification, and T cell differentiation. Survival analysis (Figures 6I,J) revealed that patients with high-ubiquitination endothelial cells had shorter OS and PFS, suggesting that endothelial cell ubiquitination might be linked to poor prognosis in HCC. Figure 6K presents key ubiquitination-related genes (e.g., UBE2E2, UBE2J2, ATXN3, UBE2I) that were significantly upregulated in high-ubiquitination endothelial cells, emphasizing their potential role in regulating metabolism, signaling, and immune functions, potentially affecting tumor progression and patient prognosis.

### 3.7 EMT cell ubiquitination in tumor microenvironment

**FIGURE 7:**
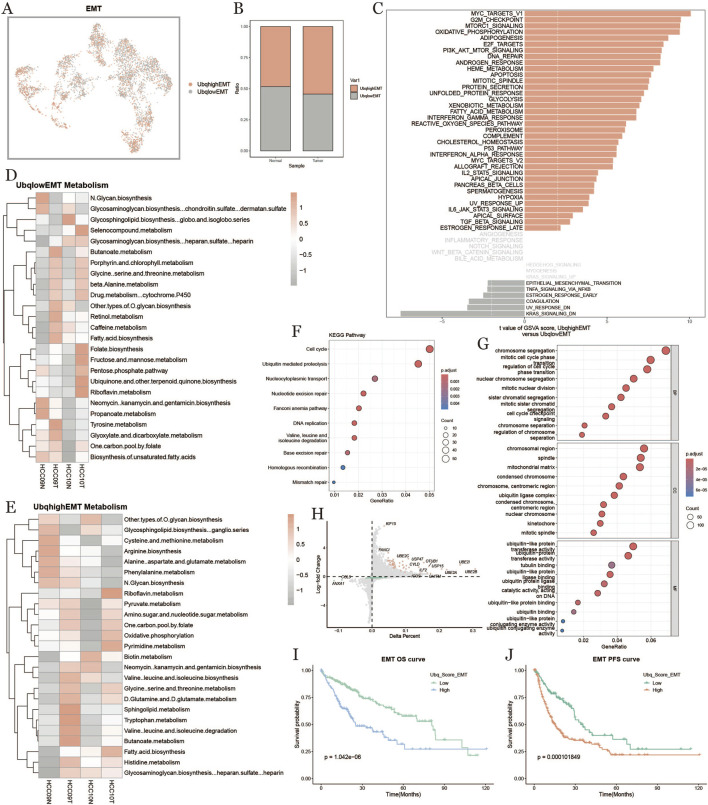
Characterization of effector memory T cells based on ubiquitination level. **(A)**: EMT distribution map based on the difference of ubiquitination level. **(B)**: Percentage of EMT cells with high and low ubiquitination levels in normal and tumor groups. **(C)**: Comparison of EMT cell pathways with high and low ubiquitination levels in tumor tissues. **(D, E)**: Metabolic heatmap of high and low ubiquitination levels of EMT cells. **(F)**: KEGG enrichment. **(G)**: Results of the analysis of KEGG enrichment. **(G)**: GO enrichment analysis result graph. **(H)**: Diagonal dot plot of differential genes of EMT cells with high and low ubiquitination levels in tumor tissues. **(I, J)**: Survival KM curves of overall survival and progression-free survival of EMT cells under the difference of ubiquitination levels.

To investigate the ubiquitination characteristics of epithelial-mesenchymal transition (EMT) cells in the HCC tumor microenvironment, we categorized EMT cells into high-expression (Ubqhigh EMT) and low-expression (Ubqlow EMT) groups based on their ubiquitination scores (Figure 7A). The results demonstrated that EMT cells within tumors exhibited significantly higher levels of ubiquitination compared to those in normal tissues (Figure 7B). GSVA enrichment analysis (Figure 7C) linked ubiquitination to a range of biological processes, including metabolism, cell cycle regulation, and inflammation. Metabolic pathway analysis (Figures 7D,E) revealed distinct enrichments in metabolic pathways: the low-ubiquitination group showed activity in glycosaminoglycan and fatty acid metabolism, while the high-ubiquitination group was enriched in amino acid, carbohydrate, and nucleotide metabolism. KEGG pathway analysis (Figure 7F) indicated that high-ubiquitination EMT cells were particularly enriched in cell cycle regulation, ubiquitin-mediated protein degradation, DNA replication, and repair pathways, highlighting the critical role of ubiquitination in EMT cell proliferation and genomic stability.GO enrichment analysis (Figure 7G) further highlighted ubiquitination’s importance in cell division and genome regulation, with processes like chromosome segregation, microtubule binding, spindle assembly, and DNA repair prominently featured. The volcano plot (Figure 7H) showed differential gene expression, with UBE2C and UBE2S significantly upregulated in high-ubiquitination EMT cells. Survival analysis (Figures 7I,J) indicated that patients with high-ubiquitination EMT cells had significantly lower OS and PFS, suggesting that EMT cell ubiquitination status might correlate with poor prognosis in HCC. Figure 7J shows key ubiquitination-related genes upregulated in high-ubiquitination EMT cells, further emphasizing the specific role of ubiquitination in EMT cells.

### 3.8 Spatial transcriptomics and ubiquitination in HCC

**FIGURE 8:**
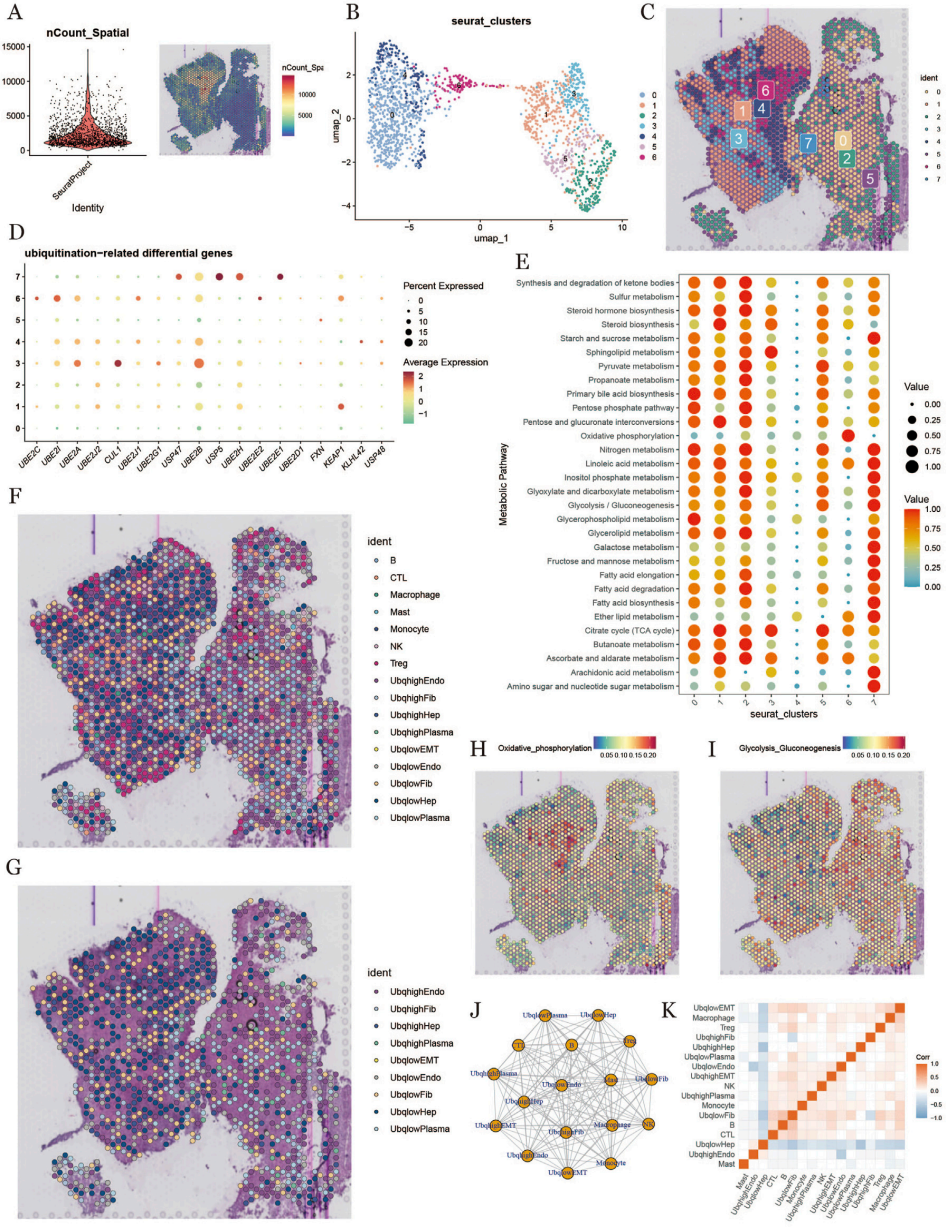
Differential expression and prognostic analysis of UBE2C. **(A)**: Forest plot of hazard ratios and 95% confidence intervals for multiple genes, the vertical dashed line at hazard ratio = 1 serves as a reference line and indicates no effect. Hazard ratios to the right indicate increased risk, and those to the left indicate decreased risk. **(B)**: Intersection of gene sets taken for each survival stage. **(C)**: Differences in UBE2C expression between tumor and normal tissues in the TCGA cohort. **(D)**: Analysis of the paired differences in UBE2C expression between tumor and normal tissues in the TCGA cohort. **(E)**: Differences in UBE2C expression in TCGA cohort at high/low tumor grades. **(F)**: Differences in UBE2C expression at various stages and prognostic analysis in the TCGA cohort. **(G)**: Differences in UBE2C expression at high/low tumor grades.UBE2C expression differences in each stage. **(G)**: UBE2C expression differences in tumor vs. normal group in GEO dataset. **(H)**: Number of surviving vs. dead samples with different UBE2C expression levels performing chi-square test. **(I)**: Kaplan-Meier survival analysis of three survival stages (OS, DSS, PFI). **(J)**: Kaplan-Meier survival of the four patient groups analysis, where Q1 represents the 25% of samples with the highest expression and Q4 represents the 25% of samples with the lowest expression. **(K)**: meta-analysis of single-factor cox survival analysis for multiple datasets.

To further explore the ubiquitination characteristics in HCC, we conducted deconvolution analysis on spatial transcriptomics data. We downloaded spatial transcriptome sequencing data (GSM6177612) from HCC tumor tissue sections derived from primary HCC tumor areas. After performing dimensionality reduction and clustering of the spatial transcriptomic data, we used UMAP for visualization, which generated seven distinct cell clusters (Figures 8A,B). Figure 8C shows the spatial distribution of all the identified cell clusters.We evaluated ubiquitination-related gene scores for each cluster (Figure 8D) and analyzed metabolic differences, finding that clusters 0, 1, 2, and 7 exhibited high metabolic activity (Figure 8E). The deconvolution analysis provided single-cell annotation results at the spatial level (Figures 8F,G). We further analyzed the intensities of glycolytic and oxidative phosphorylation pathways in different regions (Figures 8H,I) and examined spatial cell proximity relationships, revealing cell interaction signals at the spatial transcriptomic level (Figures 8J,K).

### 3.9 Prognostic value of ubiquitination-related genes in HCC

**FIGURE 9:**
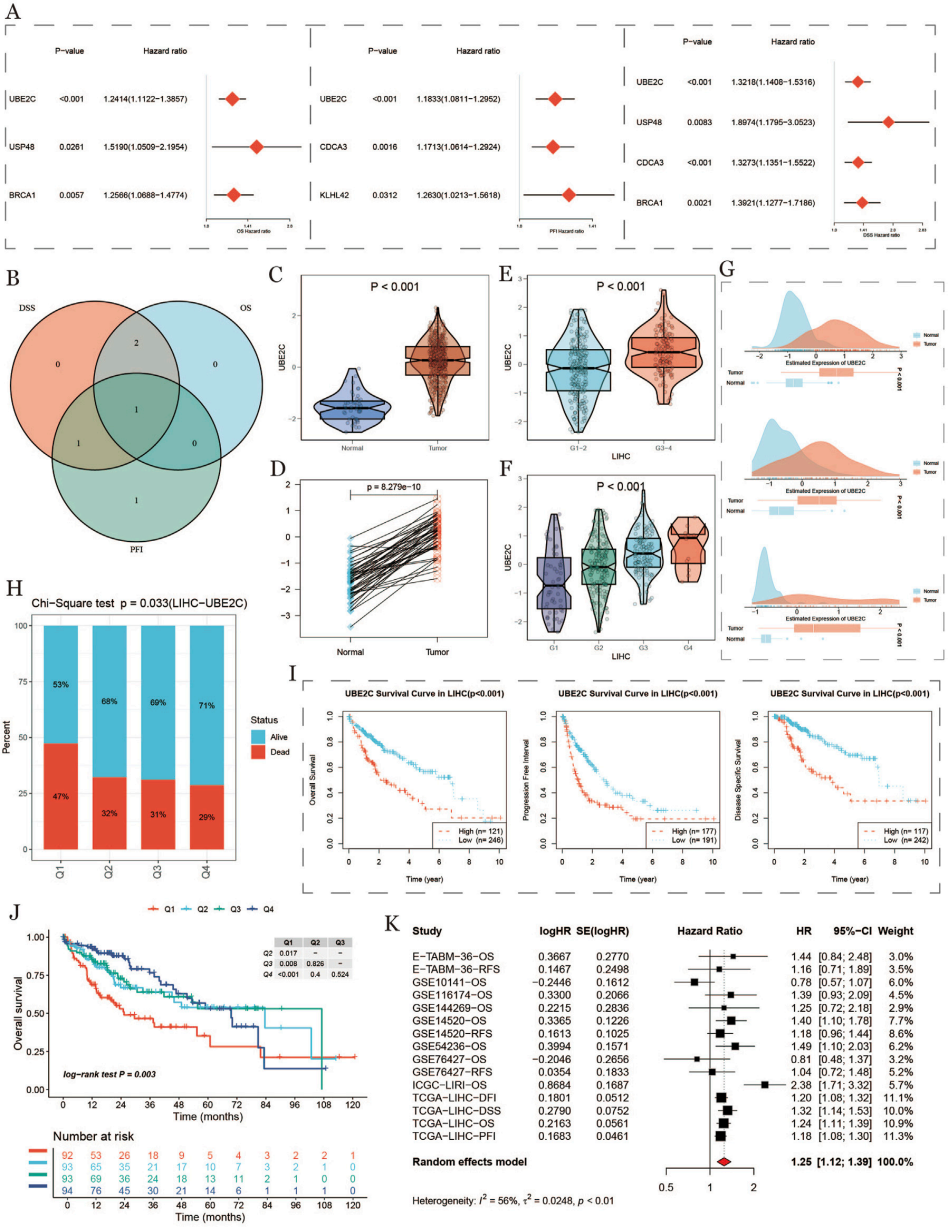
Expression differences and prognostic analysis of UBE2C **(A)** Forest plot with hazard ratios and 95% confidence intervals for multiple genes, with the vertical dashed line at hazard ratio=1 as a reference line, indicating no effect. A hazard ratio to the right indicates an increased risk, while a left hazard ratio indicates a reduced risk. **(B)**: Intersection of genes in each survival period. **(C)**: Differences in the expression of UBE2C in tumors and normal tissues in TCGA cohorts. **(D)**: Paired analysis of the expression of UBE2C in tumors and normal tissues in the TCGA cohort. **(E)**: Differences in the expression of UBE2C in high/low tumor grades in TCGA cohorts. **(F)**: Differences in the expression of UBE2C in each stage in the TCGA cohort. **(G)**: Differential expression of UBE2C in the GEO dataset between tumor and normal groups. **(H)**: Chi-square test was performed on the number of surviving and dying samples at different UBE2C expression levels. **(I)**: Kaplan-Meier survival analysis for 3 lifetimes (OS, DSS, PFI). **(J)**: Kaplan-Meier survival analysis of four groups of patients, where Q1 represents the 25% of the samples with the highest expression and Q4 represents the 25% of the samples with the lowest expression. **(K)**: Meta-analysis of multi-dataset univariate COX survival analysis.

To evaluate the prognostic significance of ubiquitination-related genes in HCC, we analyzed key ubiquitination-related gene expression in tumor *versus* normal samples, using TCGA and GEO data to assess expression levels and their relation to patient survival. Figure 9A shows the hazard ratios (HR) and p-values for various ubiquitination-related genes (e.g., UBE2C, USP48, BRCA1, CDCA3), indicating that high expression of these genes correlates with an increased risk of tumor progression, suggesting their potential role as prognostic markers in HCC. Figure 9B shows a Venn diagram comparing DSS (disease-specific survival), OS, and PFS, highlighting the significant role of UBE2C across survival metrics. Violin and paired-difference plots (Figures 9C–F) display UBE2C expression differences between normal and tumor tissues and its expression trends across stages, with UBE2C significantly upregulated in tumor and advanced-stage tissues, suggesting its association with malignancy in HCC. Figure 9G shows the density distribution of UBE2C in high- and low-expression groups, further indicating its expression patterns in HCC. Survival differences based on CHMP4B expression showed a significant association between UBE2C expression levels and survival status (Figure 9H). Survival curves (Figures 9I,J) indicated shorter OS, DSS, and PFS in high UBE2C-expressing groups, supporting a link between high UBE2C expression and poor prognosis. A meta-analysis of UBE2C in various datasets (Figure 9K) using a random effects model confirmed that high UBE2C expression significantly increased HCC mortality risk (HR = 1.25), reinforcing its adverse impact on prognosis.

**FIGURE 10:**
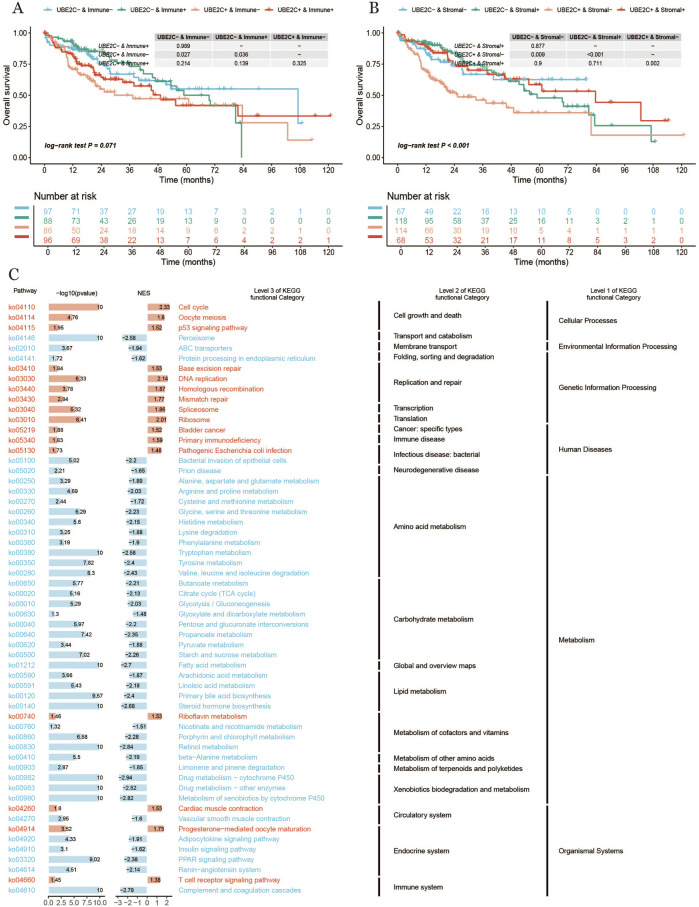
**(A, B)**: Survival curves of different subgroups of UBE2C expression **(C)** Bar graph of KEGG enrichment analysis results.

Finally, we examined the impact of UBE2C expression levels in conjunction with the activity of different cellular components—specifically immune and stromal cells—on patient survival outcomes. As illustrated in Figure 10A, the overall survival (OS) curves reveal a distinct pattern: Patients with high UBE2C expression and low immune activity exhibited the lowest survival rates, whereas those with low UBE2C expression and high immune activity demonstrated significantly higher survival. This suggests that a combination of high UBE2C expression and low immune activity may serve as a robust indicator of poor prognosis.Similarly, Figure 10B presents the OS curves based on UBE2C expression and stromal cell activity. Here, patients with high UBE2C expression and high stromal activity had the lowest survival, while those with low UBE2C expression and low stromal activity had higher survival rates. This highlights the potential prognostic significance of UBE2C in conjunction with stromal activity, further underscoring the complex interplay between UBE2C, immune cells, and stromal cells in influencing patient outcomes (Figure 10C).

### 3.10 Downregulation of UBE2C expression level significantly inhibited the proliferation, invasion and migration of hepatocellular carcinoma cells

**FIGURE 11:**
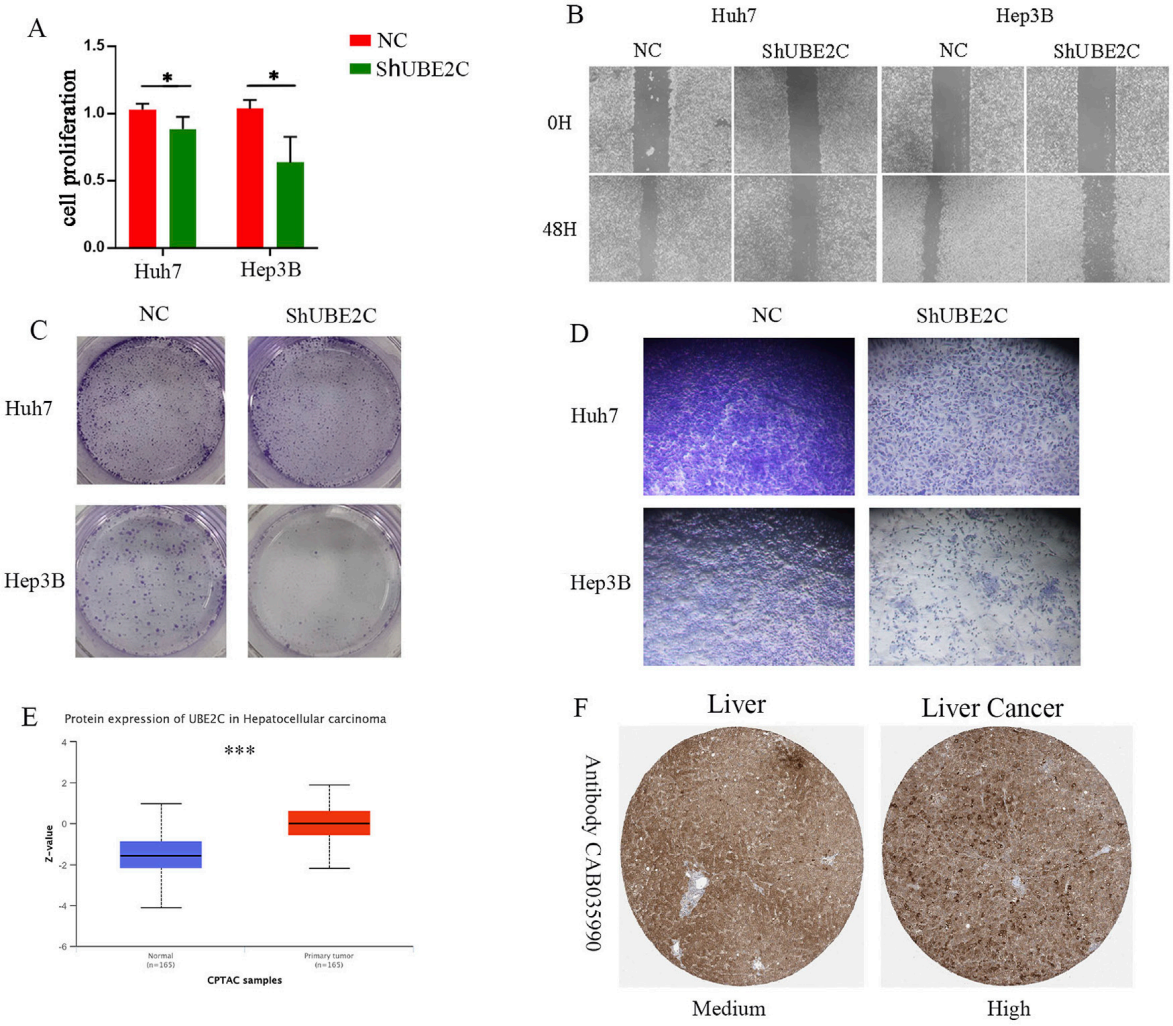
*In vitro* experiments to validate the role of UEB2C in hepatocellular carcinoma. **(A)** CCK8 assay for cell viability of UEB2C. **(B)** Wound healing assay. **(C)** Plate cloning assay. **(D)** Transwell assay **(F)** Transwell assay. **(E)** CTPAC database to verify the protein expression of UEB2C. **(F)** Protein expression of UEB2C verified by IHC. * denotes p-value less than 0.05, *** denotes p-value less than 0.001.

Considering the importance of UBE2C, we verified its role in hepatocellular carcinoma through a series of *in vitro* experiments. First, we reduced the expression of UBE2C and showed that knockdown of UBE2C significantly inhibited the activity of hepatocellular carcinoma cells by CCK8 assay (Figure 11A). To investigate the relationship between UBE2C and hepatocellular carcinoma migration, we performed a wound healing assay and showed that knockdown of UBE2C significantly inhibited the invasive migration of these cells (Figure 11B). To investigate the correlation between UBE2C and hepatocellular carcinoma proliferation, we performed a plate cloning assay, and the results showed that knockdown of UBE2C significantly inhibited the proliferative ability of hepatocellular carcinoma cells (Figure 11C).Transwell assay also showed that UBE2C enhanced the invasive migration of tumor cells (Figure 11D). Finally, CPATC database and immunohistochemical analysis confirmed elevated protein expression of UBE2C in these tissues (Figures 11E,F). In conclusion, UBE2C enhances the invasive migration of hepatocellular carcinoma cells and correlates with the malignant features of hepatocellular carcinoma.

## 4 Discussion

In recent years, ubiquitination, a crucial post-translational modification, has garnered increasing attention for its role in liver cancer (HCC) (Lu et al., 2023; Hu et al., 2021; Liu F. et al., 2024). Ubiquitination regulates protein stability, activity, and subcellular localization by tagging target proteins with ubiquitin chains, playing a pivotal role in various biological processes, including cell cycle regulation, apoptosis, metabolic reprogramming, and DNA repair (Sun et al., 2020; Cai et al., 2018). The ubiquitin-proteasome system (UPS) is a key pathway in many cancer cells, responsible for degrading tumor suppressor proteins and promoting oncogene expression, making abnormalities in ubiquitination a potential driver of HCC cells’ resistance to conventional treatments (Liao et al., 2020). Dysfunctions in deubiquitinating enzymes (DUBs) and E3 ligases in HCC cells can lead to resistance to chemotherapy and targeted therapies (Fang et al., 2023).

Our study systematically analyzed the role of ubiquitination in the HCC tumor microenvironment, focusing on its expression characteristics across different cell types (e.g., plasma cells, fibroblasts, endothelial cells, EMT cells) and its association with patient survival. Ubiquitination exhibited distinct functions in various cell types. For example, plasma cells with high ubiquitination showed significant activity in ribosome biogenesis, protein processing, and metabolic pathways, suggesting that ubiquitination may support HCC cell growth and survival through these biological processes (Tang et al., 2022; Yang et al., 2023). Similarly, active ubiquitination in fibroblasts may facilitate tumor dissemination and invasion by promoting cell proliferation and matrix remodeling. Moreover, the ubiquitination status of endothelial and EMT cells was closely linked to cell cycle regulation and DNA repair pathways, indicating that ubiquitination may promote HCC progression in these cells by enhancing cell proliferation and genome stability. Ubiquitination participates in the process of angiogenesis by affecting the proliferation, migration, and lumen formation of endothelial cells. E3 ubiquitin ligases, such as ID1 (inhibitor of differentiation 1), may regulate angiogenesis by modulating the cell cycle of endothelial cells and signaling pathways related to VEGF (vascular endothelial growth factor). Additionally, ubiquitination plays an important role in the matrix remodeling of endothelial cells, contributing to the stabilization and maturation of newly formed blood vessels.These findings highlight the multi-level regulatory role of ubiquitination within the HCC tumor microenvironment, mediating various signaling pathways and biological processes across different cell types (Villalba et al., 2013). Among ubiquitination-related genes, UBE2C emerged as a significant prognostic predictor. UBE2C, a key E2 ubiquitin-conjugating enzyme, was notably upregulated in HCC tissues and strongly associated with poor patient prognosis. Our subgroup analysis revealed that high UBE2C expression, in combination with low immune activity or high stromal activity, significantly decreased survival rates. This suggests that UBE2C may promote tumor progression by inhibiting anti-tumor immune responses and enhancing stromal cell activity (Zhang et al., 2018; Yuan et al., 2022).

Additionally, pathway enrichment analysis revealed that UBE2C is involved in several critical pathways related to tumor growth and progression, including cell cycle regulation, p53 signaling, DNA damage repair, and metabolic control. These pathways are crucial for tumor cell proliferation, genomic stability, and metabolic reprogramming, further underscoring the central role of UBE2C in the development of HCC (He et al., 2023). Immune analysis also suggested that UBE2C may promote HCC progression through multiple mechanisms. Overexpression of UBE2C could suppress the anti-tumor immune response, impairing immune cells’ ability to recognize and eliminate tumor cells, thus allowing tumor cells to evade immune surveillance (Li et al., 2020). Moreover, high UBE2C expression in stromal cells was linked to the remodeling of the tumor microenvironment, suggesting that UBE2C may promote angiogenesis and matrix remodeling by modulating the activity of fibroblasts and endothelial cells, ultimately driving tumor invasion and metastasis (Jin et al., 2020). These findings indicate that UBE2C may serve as a promising therapeutic target in the treatment of hepatocellular carcinoma (HCC). Given its high specificity in the ubiquitination process as an E2 ubiquitin-conjugating enzyme, it is feasible to develop inhibitors that specifically target the active site of UBE2C. This targeted approach can minimize non-specific effects on other cellular functions, thereby enhancing the efficacy of the treatment. Furthermore, considering UBE2C’s significant role in modulating the activity of immune and stromal cells within the HCC microenvironment, combination therapies that incorporate immune checkpoint inhibitors or stromal-targeting agents may synergistically augment the effectiveness of UBE2C inhibitors.

Despite highlighting the critical role of ubiquitination in HCC, our study has several limitations. Although this research utilizes data from public databases, providing a relatively large sample size, the substantial heterogeneity among HCC patients may affect the generalizability of our findings. Variations in tumor characteristics and microenvironmental conditions across patients could lead to differences in how ubiquitination impacts HCC progression (Raevskiy et al., 2023). Additionally, public database data often lack key clinical information, such as detailed disease progression and treatment history, which may limit the accuracy and clinical relevance of our analysis (Fan Y. et al., 2024; Pan et al., 2024). Moreover, given that ubiquitination is a dynamic and highly complex regulatory mechanism, future studies should consider employing proteomic approaches, such as mass spectrometry, to directly assess ubiquitination levels, offering a more precise evaluation of its role in HCC.

## 5 Conclusion

In this study, we systematically investigated the role of ubiquitination in the tumor microenvironment of hepatocellular carcinoma (HCC), revealing its expression characteristics in different cell types and its relationship with patient prognosis. The findings suggest that the role of ubiquitination in hepatocellular carcinoma progression is not only limited to the regulation of cell cycle, apoptosis and metabolic pathways, but also promotes tumor growth and metastasis by influencing tumor cell proliferation, invasion and immune escape. For example, highly ubiquitinated fibroblasts may promote tumor spread by promoting cell proliferation and stromal remodeling, while highly ubiquitinated endothelial and epithelial-mesenchymal transition (EMT) cells promote HCC progression by regulating cell cycle and DNA repair pathways.

## Data Availability

The data presented in the study are deposited in the GEO database (https://www.ncbi.nlm.nih.gov/geo/), specifically the dataset GSE149614, which contains sequencing data from 10 hepatocellular carcinoma (HCC) patients. We selected nontumor and primary tumor samples for analysis. Spatial transcriptomics data were obtained from the primary HCC tissue section GSM6177612. Additionally, RNA-seq data for pancreatic cancer, comprising 424 samples and associated survival data, were acquired from the TCGA cohort via the UCSC Xena platform (https://xena.ucsc.edu/) for survival analysis. We retrieved a set of 78 ubiquitination-related genes from the GO database (https://geneontology.org/).
